# Semi-nonparametric modeling of topological domain formation from epigenetic data

**DOI:** 10.1186/s13015-019-0142-y

**Published:** 2019-03-05

**Authors:** Emre Sefer, Carl Kingsford

**Affiliations:** 1 Machine Learning Department, Carnegie Mellon University, 5000 Forbes Avenue, Pittsburgh, 15213 USA; 2 Computational Biology Department, Carnegie Mellon University, 5000 Forbes Avenue, Pittsburgh, 15213 USA

**Keywords:** Chromatin, Conformation, Capture, Topological domains, Epigenetic modifications, Prediction

## Abstract

**Background:**

Hi-C experiments capturing the 3D genome architecture have led to the discovery of topologically-associated domains (TADs) that form an important part of the 3D genome organization and appear to play a role in gene regulation and other functions. Several histone modifications have been independently associated with TAD formation, but their combinatorial effects on domain formation remain poorly understood at a global scale.

**Results:**

We propose a convex semi-nonparametric approach called *nTDP* based on Bernstein polynomials to explore the joint effects of histone markers on TAD formation as well as predict TADs solely from the histone data. We find a small subset of modifications to be predictive of TADs across species. By inferring TADs using our trained model, we are able to predict TADs across different species and cell types, without the use of Hi-C data, suggesting their effect is conserved. This work provides the first comprehensive joint model of the effect of histone markers on domain formation.

**Conclusions:**

Our approach, *nTDP*, can form the basis of a unified, explanatory model of the relationship between epigenetic marks and topological domain structures. It can be used to predict domain boundaries for cell types, species, and conditions for which no Hi-C data is available. The model may also be of use for improving Hi-C-based domain finders.

## Background

The emerging evidence suggests that 3D nuclear architecture is important for the regulation of gene expression and it is tightly linked to the function of the genome. For instance, expression in the beta-globin locus is mediated by folding to bring an enhancer and associated transcription factors within close proximity of a gene [1, 2]. Similarly, loci of mutations that affect expression of genomically far-away genes (eQTLs) interact significantly more frequently within a contact range in 3D to their regulated genes [3], indicating that 3D genome structure plays a wide-spread role in gene regulation. Lastly, spatial regions that interact with nuclear lamina are generally inactive [4]. Measuring and modeling the 3D shape of a genome is thus essential to obtain a more complete understanding of how cells function.

Chromatin interactions obtained from a variety of recent chromosome conformation capture experimental techniques such as Hi-C [5] have resulted in significant advances in our understanding of the geometry of chromatin structure [6, 7]. These experiments yield matrices of counts that represent the frequency of cross-linking between restriction fragments of DNA at a certain resolution. Analysis of the resulting matrix by Dixon et al. [8] led to the discovery of topologically-associated domains (TADs) which correspond to consecutive, highly-interacting matrix regions typically a few megabases in size that are close in densely packed chromatin. TADs have been identified across different cell cycle phases and in prokaryotes [9]. Several lines of evidence suggest that TADs are a building block of genomic regulatory architecture [10, 11]. Segmental packaging of genome via TADs likely have critical roles in cell dynamics such as long-range transcriptional regulation and cell differentiation [12, 13].

The mechanism by which these TADs form and are demarcated is still largely unknown. A plethora of epigenetic modifications have been identified in metazoan genomes that are associated with 3D genome shape [14], and thus TADs. Several modifications have been found to be specifically correlated with TAD boundaries [8]. For instance, histone modifications H3K4me3m H3K27ac and insulator proteins are enriched within TAD boundaries [15], although the causal direction of these associations is still unknown [12]. Despite these analyses, the complete picture of how histone modifications are related to TAD formation is missing. This is partially because previous analyses relating histone marks to domain boundaries have often considered each histone mark independently, without accounting for their combined affects. It is unknown to what extent relationships between the histone markers are important or whether there is a small set of markers that are of primary importance.

Here, we develop and train a joint model, which we call ***nTDP*** **(Nonparameteric Topological Domain Partitioner)**, of how histone modifications are associated with domain boundaries and interiors. We show that we are able to train this model optimally in polynomial time because its likelihood function is convex. The model does not make any assumptions about the effect of each histone mark on domain formation, and instead fits the histone-domain relationship nonparametrically. Using this model, we systematically identify a small set of histone markers that in combination appear to explain TAD boundaries. We find a small number of epigenetic elements account for a large proportion of the accuracy of TAD prediction. All of these identified marks fail to predict domain boundaries when considered independently. We show that these markers are conserved across species and cell types in a very strong way: models trained on mouse continue to work well on human, and models trained on IMR90 cells continue to work on embryonic stem cells.

Our approach, *nTDP*, can form the basis of a unified, explanatory model of the relationship between epigenetic marks and topological domain structures. It can be used to predict domain boundaries for cell types, species, and conditions for which no Hi-C data is available. The model may also be of use for improving Hi-C-based domain finders. The rest of the paper is organized as follows: We start by formally defining *nTDP* model. Then, we present provably optimal methods to train our model from markers and TADs, as well as to predict TADs over trained model. Lastly, we present results on prediction of domains on the same species as well as across species and cell types.

### Related work

Previous work mainly focused on analyzing epigenetic data in an unsupervised way. Segway [16] and ChromHMM [17] take as input a collection of genomics datasets and learn chromatin states that exhibit similar epigenetic activity patterns which then have different interpretations such as transcriptionally active, Polycomb-repressed. Libbrecht et al. [18] improve Segway predictions by integrating Hi-C data which is not as abundant as histone data, whereas [19] jointly infers chromatin state maps in multiple genomes by a hierarchical model. However, none of these methods deal directly with TADs. Even though a subset of their chromatin states overlap with TADs, predicting TADs from them heuristically does not perform well. Additionally, they either ignore the histone densities, or make parametric distribution assumptions such as geometric or normal which are not always reflected in the true data. When modified to run in a supervised setting, they cannot capture the most informative subset of epigenetic elements.

The recent approach [20] proposes a supervised learning method based on random forests to predict TAD boundaries from histone modifications and chromatin proteins. In general, this approach is reported to perform quite accurately in predicting boundaries. However, it does not model interior TAD segments and it treats each segment independently ignoring the fact that TADs form as a result of the joint effects of multiple segments.

## The *nTDP* model

### The likelihood function

Let *V* be the ordered set of genome restriction fragments (bins), where each bin *v* represents the interval $[vr-r+1$, *vr*], where *r* is the Hi-C resolution. Let *M* be the set of histone modifications (markers) over *V*. The marker data $H = (h_{vm})$ is a $|V| \times |M|$-matrix where its (*v*, *m*)’th entry $h_{vm}$ is the count of the occurrences of marker *m* inside segment *v*. Let $d=[s, e]$ be a domain (interval) where *s* and *e* are its start and end boundaries respectively, $\{s+1, \ldots , e-1\}$ are the segments inside *d*, and let $D = \{[s_{1}, e_{1}], [s_{2}, e_{2}], \ldots , [s_{i}, e_{i}] \}$ be a partition of *V* where none of the domains overlap.

We propose a supervised, semi-nonparametric, high-dimensional model *nTDP* that uses *H* to model and predict *D*. Our model can be seen as a generalization of Conditional Random Field [21, 22] where we have continuous weights instead of binary features and where we model the marker effects semi-nonparametrically.

Specifically, we assume there are 3 types of segments in *V* that are relevant for modeling: those that are at the domain boundaries ($V_{\mathbf {b}}$), those that are in the interior of domains ($V_{\mathbf {i}}$), and those that are not part of a domain ($V_{\mathbf {e}}$), and we have $V = V_{\mathbf {b}}\cup V_{\mathbf {i}} \cup V_{\mathbf {e}}$. For each marker type *m*, we have 3 types of *effect functions*, $f^b_m(c, \mathbf {w_m^b}), f_m^i(c, \mathbf {w_m^i}), f_m^e(c, \mathbf {w_m^e})$, that will describe the relationship between marker count *c* and the fragment type (b, i, e) for marker type *m*. Here, $\bf {w_m^b},\bf {w_m^i},\bf {w_m^e}$ are parameters that we will fit to determine the shape of the effect function. Thus, for example, $f_m^i(c, \mathbf {w_m^i})$ will describe how a count of *c* for marker *m* influences whether the fragment is in the interior (i) of a domain.

We assume that these effect functions combine linearly. Therefore, let

1 $$\begin{aligned} E^{b}_{vq} = \sum _{m \in M} f^{b}_{m}(c^{q}_{vm}, \mathbf {w^{b}_{m}}) \end{aligned}$$

be the total effect of all the markers on fragment *v* for boundary formation (b). Summations $E^{i}_{vq}$ and $E^{e}_{vq}$ are defined analogously for interior (i) and inter-domain fragments (e).

Let *W* be the union of model parameters $\mathbf {w_m^b}, \mathbf {w_m^i}, \mathbf {w_m^e}$, and let $D^\text {train} = \{D^q : q = 1,\dots ,Q\}$ be several domain decompositions (in different sequences or conditions) and let $H^\text {train} = \{H^q : q = 1,\dots , Q\}$ be a set of corresponding histone markers. Under the assumption that the training pairs are independent, the log-likelihood of parameters *W* given $D^\text {train}$ is

2 $$\begin{aligned} \log \left (P(D^{\text {train}} | W, H^{\text {train}})\right ) = \sum _{q}\log (P(D^{q} | W, H^{q}) ). \end{aligned}$$

We define the probability $P(D^{q}, W, H^{q}) = \frac{\exp ^{F(D^{q}, W, H^{q})}}{\sum _{F^{'}} \exp ^{F^{'}}}$ where $F(D^{q}, W, H^{q})$ is the total quality of partition $D^{q}$ and marker data $H^{q}$ under model parameters *W*. Let $V^{q}$ be the set of segments in pair *q*. Due to independence of segments:

3 $$\begin{aligned}\log \left (P(D^{q} | W, H^{q})\right) = \overbrace{\sum _{d=[s, e] \in D^{q}} \left( \sum _{v \in \{s, e\}} \overline{c}_{b} E^{b}_{vq} + \sum _{v =s+1}^{e-1}\overline{c}_{i} E^{i}_{vq} \right) \, + \sum _{v \in V^{q}_{e}} \overline{c}_{e} E^{e}_{vq}}^{\log \left( P(D^{q} , W, H^{q})\right) = F(D^{q}, W, H^{q})} \nonumber & -\log (Z^{q}_{|V^{q}|}) \end{aligned}$$

where $Z^{q}_{|V^{q}|} = \sum _{D'}\,P(D' , W, H^{q})$ is the partition function defined over all possible nonoverlapping partitions $D'$, $\overline{c}_{b}, \overline{c}_{i}, \overline{c}_{e}$ are relative weights of different types of fragments to account for unbalanced training set, and $V_e^q$ is the set of fragments that do not belong to any domain in $D^q$.

### Nonparametric form of the effect functions

Because the shape of the marker effect function is unknown, we choose the *f* functions from the nonparametric family of Bernstein basis polynomials. Bernstein polynomials can approximate any effect function and additionally can handle imposed shape constraints such as monotonicity and concavity.

Let *A* be the chosen dimension of these polynomials; larger *A* results in a more expressive family, but more parameters to fit. Let $m_{\max }$ be the maximum possible density of marker *m*. This is is used to transform the input $c^{q}_{vm}$ to the range [0, 1]; therefore define $p^{q}_{vm} = {c^{q}_{vm}}/{m_{\max }}$. We model $f^{b}_{m}(c^{q}_{vm}, \mathbf {w^{b}_{m}})$ for segment *v* by a Bernstein polynomial $B_{A}(p^{q}_{vm}, \mathbf {w^{b}_{m}})$ as in:

4 $$\begin{aligned} f^{b}_{m}(c^{q}_{vm}, \mathbf {w^{b}_{m}}) = B_{A}\left( p^{q}_{vm}, \mathbf {w^{b}_{m}} \right) = \sum _{i=0}^{A} w^{b}_{m}[i] \overbrace{ {A \atopwithdelims ()i} \left( p^{q}_{vm}\right) ^{i} \left( 1-p^{q}_{vm}\right) ^{A-i}}^{b_{i, A} (p^{q}_{vm})} \end{aligned}$$

where $b_{i, A} (p^{q}_{vm})$ are the base Bernstein kernels.

## Optimal algorithms for training and inference

We must train the parameters *W* for the above model using data of the form $D^\text {train}, H^\text {train}$. We will examine these trained parameters (and several good solutions for them) for insights into which markers are most informative for describing $D^\text {train}$ and thus topological domains.

### **Problem 1**

*Training*: Given a set of marker data $H^{\text {train}}$, likely from several chromosomes and cell conditions, and corresponding set of TAD decompositions $D^{\text {train}}$, we estimate the most likely parameters *W* according to Eq. 2.

### **Problem 2**

*Inference*: Given marker data *H* model parameters *W*, we estimate the best domain partition *D* of the track.

### Training

A nice feature of the objective (3) is that it is convex in its arguments, $\{\mathbf {w^{b}_{m}}, \mathbf {w^{i}_{m}},$ $\mathbf {w^{e}_{m}}\}_{m \in M}$, which follows from linearity, composition rules for convexity, and convexity of the negative logarithm. However, training involves several challenges: (a) computing the partition function $Z^{q}_{|V^{q}|}$ in (3), and (b) estimating *W* so that the weights are sparse. We solve each of these challenges next.

#### Estimating the partition function

We estimate $Z^{q}_{|V^{q}|}$ in (3) recursively in polynomial time since each segment can belong to one of 4 states: domain start (*sb*), inside a domain (*i*), domain end (*eb*), non-domain (*e*), and state of each segment depends only on the previous segment’s state. Let $Y = \{sb, i, eb, e\}$, and $Z_{|V^{q}|}^{q} = Z^{q}_{|V^{q}|, eb}\,+\,Z^{q}_{|V^{q}|, e}$ which components can be estimated by:

5 $$\begin{aligned} Z^{q}_{v, x} = \sum _{y \in Y} Z^{q}_{v-1, y} T_{y, x} \exp ^{E^{x}_{vq}} \end{aligned}$$

where $Z^{q}_{v,sb}$, $Z^{q}_{v,i}$, $Z^{q}_{v,eb}$, $Z^{q}_{v,e}$ represent the partition function up to segment *v* ending with *sb*, *i*, *eb* and non-domain respectively. *T* is a $4 \times 4$ binary state transition matrix where $T_{y, x} = 1$ if a segment can be assigned to *x* given previous segment is assigned to state *y* such as $(y, x) \in \{(sb, i), (sb, eb), (i, i), (i, eb), (eb, sb)$, $(eb, e), (e, sb), (e, e)\}$, otherwise 0. Initial conditions are $Z^{q}_{0,sb} = Z^{q}_{0,i} = Z^{q}_{0,eb} = 0$, $Z^{q}_{0,e} = 1$. To avoid overflow in estimating $Z^{q}_{|V^{q}|}$ and speed it up, we estimate $\log (Z^{q}_{|V^{q}|})$ by expressing it in terms of log of the sum of exponentials and forward and backward variables ($\alpha$, $\beta$) similar to Hidden Markov Model [21].

#### Estimating a sparse set of good histone effect parameters

We would like to augment objective function (2) so that we select a sparse subset of markers from the data and avoid overfitting. If the coefficients $\mathbf {w^{b}_{m}} = 0$, then there is no influence of marker *m*. For this purpose, we will impose grouped lasso type of regularization on the coefficients $w_{mk}$. Grouped lasso regularization has the tendency to select a small number of groups of non-zero coefficients but push other groups of coefficients to be zero.

We introduce two types of regularization. First, we require that many of the weights be 0 using an $L_2$-norm regularization term. Second, we want the effect functions $\{f\}$ to be smooth. Let $P = \{b, i, e\}$. We modify our objective to trade off between these goals:

6 $$\begin{aligned} \mathop {\text{argmin}}\limits _{W}\;-\sum _{q}\log \left (P(D^{q}| W, H^{q})\right )+\overbrace{\lambda _{1} \sum _{p \in P} \left ( \sum _{m \in M} \left|\left|\mathbf {w^{p}_{m}}\right|\right| \right)^{2}+\lambda _{2}\sum _{p \in P} \sum _{m \in M} R(f^{p}_{m})}^{\text {Regularization}} \end{aligned}$$

where $\lambda _{1}$, $\lambda _{2}$ are the regularization parameters, and $R(f^{p}_{m})$ is the smoothing function for effect of marker *m* at $p \in P$:

7 $$\begin{aligned} R(f^{p}_{m}) = \int _{x} \left( \frac{\partial ^{2} f^{p}_{m}(x,\mathbf {w^{p}_{m}})}{\partial x^{2}}\right) ^{2} dx \end{aligned}$$

Group lasso in (6) uses the square of block $l_{1}$-norm instead of $l_{2}$-norm group lasso which does not change the regularization properties [23]. Objective function (6) is convex which follows from the convexity of $R(f^{p}_{m})$ as proven in Theorem 1.

##### **Theorem 1**

$R(f^{p}_{m})$ *is a convex function of* $\mathbf {w^{p}_{m}}$.

##### *Proof*

Second-order derivative in (7) can be written more explicitly as in (8) according to [24]:

8 $$\begin{aligned} \frac{\partial ^{2} f^{p}_{m}(x,\mathbf {w^{p}_{m}})}{\partial x^{2}} = A(A-1) \sum _{i=0}^{A-2} (w^{p}_{m}[i+2]-2w^{p}_{m}[i+1]+w^{p}_{m}[i]) \genfrac(){0.0pt}0{A-2}{i} x^{i}(1-x)^{A-2-i} \end{aligned}$$

which turns $R(f^{p}_{m})$ into (9):

9 $$\begin{aligned}&\int _{0}^{1} \left( \frac{\partial ^{2} f^{p}_{m}(x)}{\partial x^{2}}\right) ^{2} dx = A^{2}(A-1)^{2} \sum _{i=0}^{A} \sum _{j=i}^{A} (w^{p}_{m}[i] w^{p}_{m}[j])\nonumber \\&\quad \left( \sum _{q=\overline{e}_{i}}^{\min (i,2)} \sum _{r=\overline{e}_{j}}^{\min (j,2)} (-1)^{q+r} \genfrac(){0.0pt}0{2}{q} \genfrac(){0.0pt}0{2}{r} T^{i-q}_{j-r}(x) \right) \end{aligned}$$

where $\overline{e}_{p} = \max (0,2-A+p)$, $T^{i-q}_{j-r}(x)$ is defined below and $\beta (i+j-q-r+1,2A-3-i-j+q+r)$ is the beta function:

10 $$\begin{aligned} T^{i-q}_{j-r}(x) = \genfrac(){0.0pt}0{A-2}{i-q} \genfrac(){0.0pt}0{A-2}{j-r} \underbrace{\int _{0}^{1} x^{i-q}(1-x)^{A-2-i+q} x^{j-r}(1-x)^{A-2-j+r} dx}_{\beta (i+j-q-r+1, 2A-3-i-j+q+r)} \end{aligned}$$

$R(f^{p}_{m})$ is quadratic function of $\mathbf {w^{p}_{m}}$. Its convexity follows from semidefiniteness of the resulting polynomial. $\square$

We note that (6) is convex, but it is a nonsmooth optimization problem because of the regularizer. We solve it efficiently by using an iterative algorithm from multiple kernel learning [23]. By Cauchy-Schwarz inequality:

11 $$\begin{aligned} \sum _{p \in P} \left ( \sum _{m \in M} \left|\left|\mathbf {w^{p}_{m}}\right|\right| \right )^{2} \le \sum _{p \in P} \sum _{m \in M} \frac{\left|\left|\mathbf {w^{p}_{m}}\right|\right|^{2}}{\gamma _{mp}} \end{aligned}$$

where $\gamma _{mp} \ge 0$, $\sum _{m \in M} \gamma _{mp} = 1, \;\, p \in P$, and the equality in (11) holds when

12 $$\begin{aligned} \gamma _{mp} = \frac{\left|\left|\mathbf {w^{p}_{m}}\right|\right|}{\sum _{m \in M} \left|\left|\mathbf {w^{p}_{m}}\right|\right|}, \quad p \in P \end{aligned}$$

This modification turns the objective into the following which is jointly convex in both $\mathbf {w^{p}_{m}}$ and $\gamma _{mp}$:

13 $$\begin{aligned}\mathop {\text{argmin}}\limits _{W}\;-\sum _{q}\log \left (P(D^{q} | W, H^{q})\right )\,+\,\sum _{p \in P} \sum _{m \in M} \left (\lambda _{1}\frac{\left|\left|\mathbf {w^{p}_{m}}\right|\right|^{2}}{\gamma _{mp}}\,+\,\lambda _{2} R(f^{p}_{m})\right ) \end{aligned}$$

14 $$\begin{aligned}&\,\text {s.t.} \;\; \sum _{m \in M} \gamma _{mp} = 1.0,\quad p \in P \end{aligned}$$

15 $$\begin{aligned}&\;\;\;\;\quad \gamma _{mp} \ge 0,\quad m \in M, p \in P \end{aligned}$$

We solve this by alternating between the optimization of $\mathbf {w^{p}_{m}}$ and $\gamma _{mp}$. When we fix $\gamma _{mp}$, we can find the optimal $\mathbf {w^{p}_{m}}$ by any quasi-newton solver such as L-BFGS [25] which runs faster than the other solvers such as iterative scaling or conjugate gradient. When we fixed $\mathbf {w^{p}_{m}}$, we can obtain the best $\gamma _{mp}$ by the closed form equation (12). Both steps iterate until convergence.

### Training extensions

We can model a variety of shape-restricted effect functions by Bernstein polynomials that cannot be easily achieved by other nonparametric approaches such as smoothing splines [24]. We add the following constraints to ensure monotonicity:

16 $$\begin{aligned} w^{b}_{m}[i] \le w^{b}_{m}[i+1], \quad i = 0, \ldots , A-1 \end{aligned}$$

which is a realistic assumption since increasing the marker density should not decrease its effect. We can also ensure concavity of the effect function by:

17 $$\begin{aligned} w^{b}_{m}[i-1] - 2w^{b}_{m}[i] + w^{b}_{m}[i+1] \le 0, \quad i = 1, \ldots , A-1 \end{aligned}$$

which has a natural diminishing returns property: the increase in the value of the effect function generated by an increase in the marker density is smaller when output is large than when it is small. Our problem is different than smoothing splines since our loss function is more complicated than traditional spline loss functions due to partition function estimation in (5) which makes it hard to directly apply the smoothing spline methods [26]. In addition, these nonnegativity and other shape constraints can be naturally enforced in our method.

We can also extend the problem to modeling multiple domain subclasses instead of a single class where domains are categorized into subclasses according to their gene-expression profiles such as TADs with highly-active genes, TADs with repressive genes, etc.

### Inferring domains using the trained model

Given marker data *H* over a single track and *W*, the inference log-likelihood is:

18 $$\begin{aligned} \mathop {\text{argmax}}\limits _{D}\;\log \left (P(D | W, H)\right ) = \sum _{d=[s, e] \in \overline{D}} r_{se} x_{se}\;+\;\sum _{v \in V} E^{e}_{v} y_{v} \end{aligned}$$

where $\overline{D} = \{[s, e]\,|\,s,e \in V, \, e-s \ge 1\}$ is the set of all potential domains of length at least 2 and $r_{se} = E^{b}_{s} + E^{b}_{e} + \sum _{v =s+1}^{e-1} E^{i}_{v}$. The intuition is that variable $x_{se}=1$ when the solution contains interval [*s*, *e*], and variable $y_{v}=1$ if *v* is not assigned to any domain. The $\log (Z_{|V|})$ term is removed during inference since it is same for all *D*. We solve (19)–(20) to find the best partition *D*:

19 $$\begin{aligned}&\mathop {\text{argmax}}\limits _{D}\; \sum _{d=[s, e] \in \overline{D}} r_{se} x_{se}\;+\;\sum _{v \in V} E^{e}_{v} \left( 1 - \sum _{[s, e] \in M[v]} x_{se} \right) \end{aligned}$$

20 $$\begin{aligned}\text {s.t.} \;\; x_{se} + x_{s'e'} \le 1\qquad \forall \; \text {domains } [s,e], [s',e'] \text { that overlap} \end{aligned}$$

where *M*[*v*] is the set of intervals that span fragment *v*. We replace $y_{v}$ in (18) with $1 - \sum _{[s, e] \in M[v]} x_{se}$ since each segment can be assigned to at most a single domain. (20) ensures that inferred domains do not overlap. Problem (19)–(20) is *Maximum Weight Independent Set* in interval graph defined over domains which can be solved optimally by dynamic programming in $O(|V|^{2})$ time.

## Results

### Experimental setup

We binned ChIP-Seq histone modification and DNase-seq data at $40~\hbox {kb}$ resolution, estimate RPKM (Reads Per Kilobase per Million) measure for each bin, and transform values *x* in each bin by $\log (x+1)$, which reduces the distorting effects of high values. In the case of 2 or more replicates, the RPKM-level for each bin is averaged to get a single histone modification file, in order to minimize batch-related differences. We convert any data mapped to hg19 (mm8) to hg18 (mm9) using UCSC liftOver tool. We define TADs over human IMR90, human embryonic stem (ES), and mouse ES cells Hi-C data [8] at 40 kb resolution after normalization by [27]. We use consensus domains from Armatus [28] as the true TAD partition by selecting threshold $\gamma$ where maximum Armatus domain size is closest to the maximum Dixon et al. [8] domain size ($\gamma =0.5$ for IMR90, $\gamma =0.6$ for human ES, and $\gamma =0.2$ for mouse ES cells).

We solved the training optimization problem by L-BFGS [25]. We use the public implementation of *Armatus* [28], and obtain histone modifications from NIH Roadmap Epigenomics [29] and UCSC Encode [30]. Code and datasets can be found at http://www.cs.cmu.edu/~ckingsf/research/ntdp. *nTDP* is reasonably fast: we train on all human IMR90 chromosomes in less than 3 h on a MacBook Pro with 2.5Ghz processor and $8\hbox {Gb}$ Ram. The iterative procedure in general converges in fewer than 10 iterations.

We prevent overfitting by following a two-step nested cross-validation which has inner and outer steps. The outer *K*-fold cross-validation, for example, trains on all autosomal human chromosomes except the one to be predicted. Within each loop of outer cross-validation, we perform $(K-1)$-fold inner cross-validation to estimate the regularization parameters.

### *nTDP* finds a small subset of modifications predictive of TADs

We identified a minimal set of histone marks that can model TADs as follows: we run *nTDP* independently on each chromosome of human IMR90 to obtain 21 sets of marks. These sets overlap significantly across all chromosome pairs (hypergeometric $p < 0.05$ for all pair-wise comparisons), and a total of 16 modifications cover all chromosomes. Despite the regularization, the weights of several of these marks are still very close to 0, so we identify a non-redundant subset of the modifications by Bayesian information criterion (BIC) [21] which penalizes model complexity more strongly.

As we increase the number of included modifications from 1 to 16, the BIC decrease nearly stops after 4 modifications, with some additional small reduction up to 6 modifications. The sets of 4 and 6 modifications that were most informative are: {H3K36me3, H3K4me1, H3K4me3, H3K9me3} and {H3K4me3, H3K79me2, H3K27ac, H3K9me3, H3K36me3, H4K20me1}. These non-redundant set of elements are preserved when we repeat this procedure between species. We find that only these 4–6 modifications are needed to accurately predict TADs.

#### These marks are common in good models

**Fig. 1:**
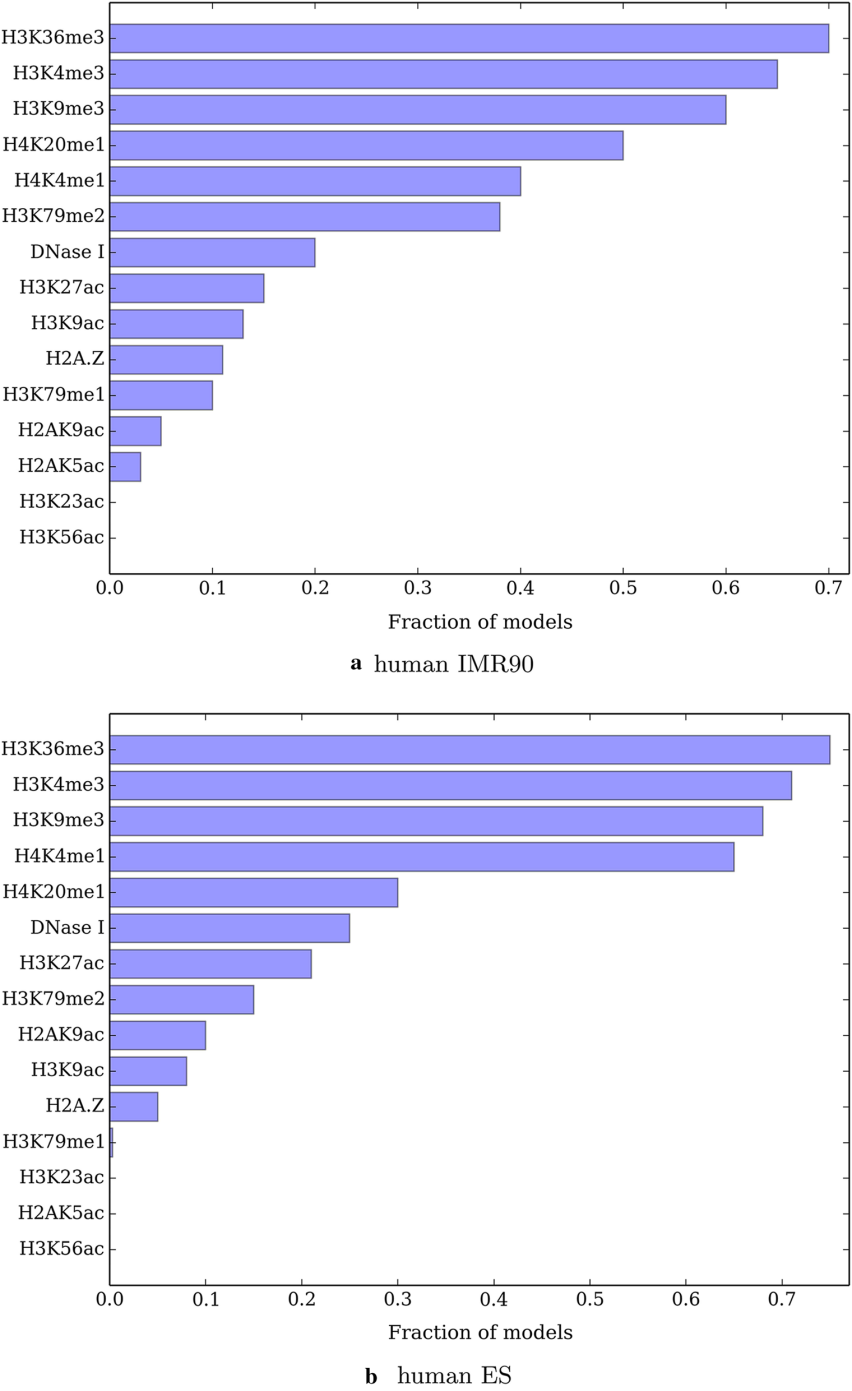
Fraction of histone modifications appearing in a best scoring four-modification model in **a** human IMR90, **b** human ES. Best scoring is defined as reaching at least $95\%$ of NVI score of the model with all modifications

**Fig. 2:**
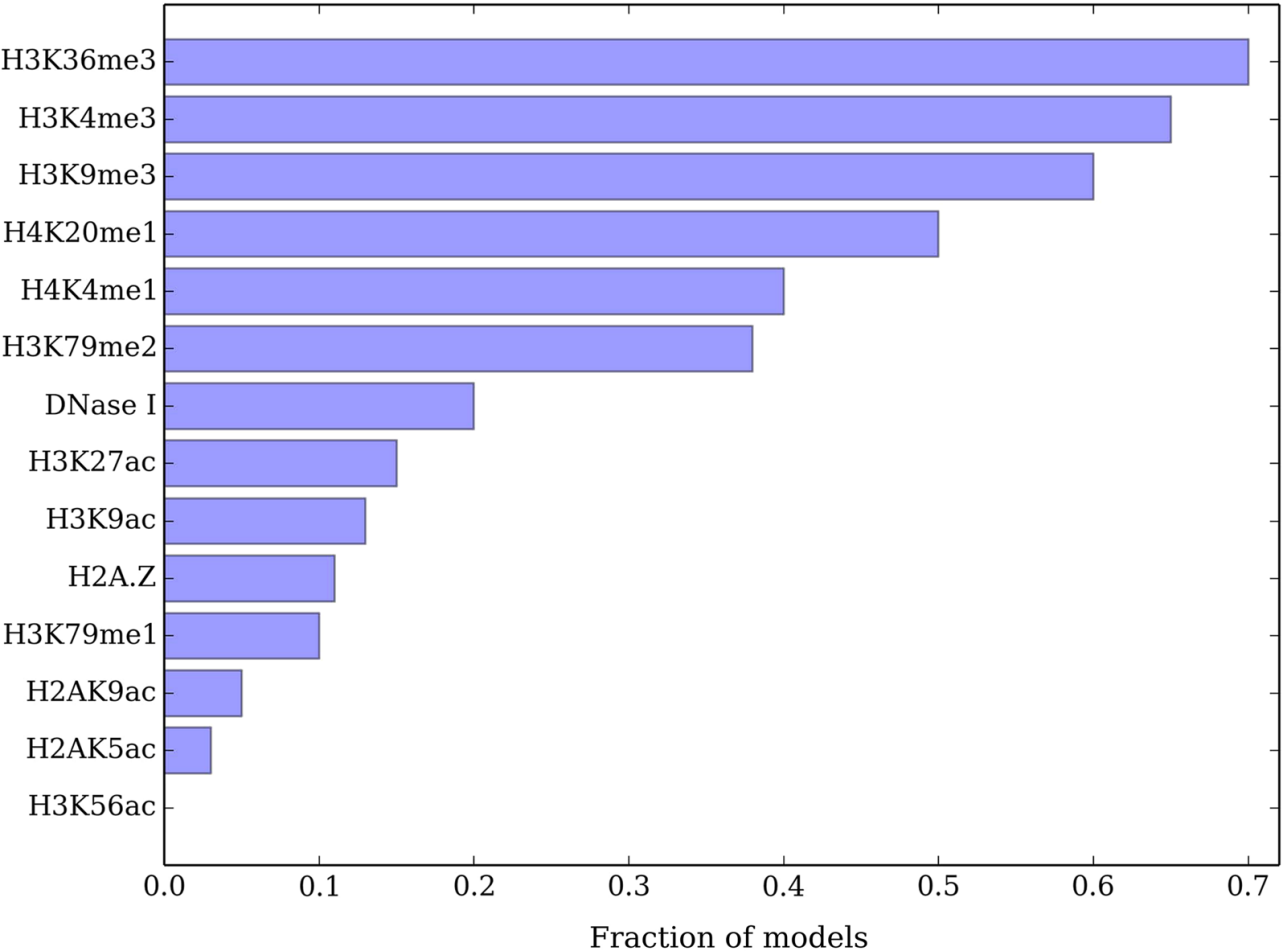
Fraction of histone modifications appearing in a best scoring four-modification parametric model in human IMR90. Best scoring is defined similar to Fig. 1

**Fig. 3:**
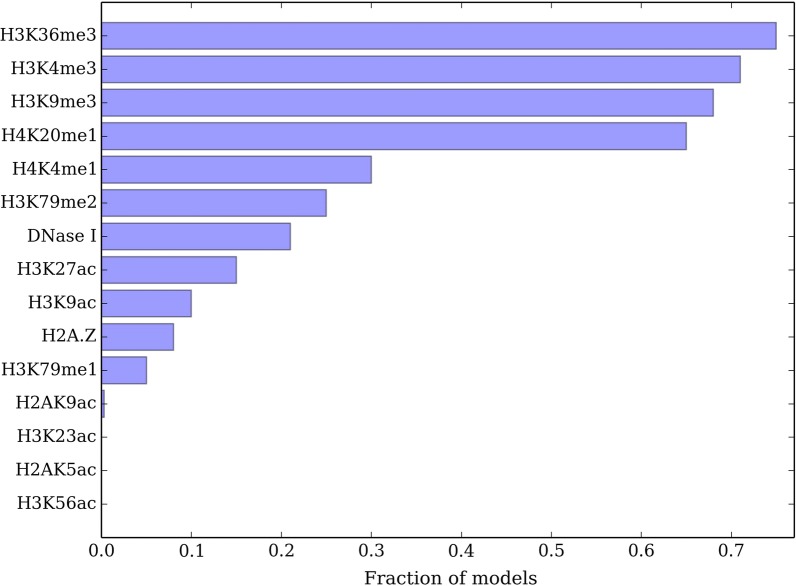
Fraction of histone modifications appearing in a best scoring four-modification model in mouse ES. Best scoring is defined similar to Fig. 1

The 4 modifications {H3K36me3, H3K4me1, H3K4me3, H3K9me3} are also enriched among a collection of high quality training solutions. We measure the agreement between estimated and true partitions by normalized variation of information $NVI=\frac{VI}{\log |V|}$ [31] where VI measures the similarity between two partitions and lower score means better performance. We analyze the fraction of models with 4 histone modifications for which NVI score is at least $95\%$ of optimum NVI score obtained by running *nTDP* over all modifications as in Fig. 1. We find 161, 139 solutions satisfying this criteria among 1820 candidates for human IMR90 and human ES histone modifications respectively. We find the 4 histone modifications above to be significantly overrepresented in the set of models for both human IMR90 and ES cells (hypergeometric $p < 0.0001$). As a boundary case, restricting the effect function to be a linear function of model parameters in human IMR90 cells does not significantly change the results as in Fig. 2. In another species, mouse ES cells, these 4 histone modifications are also the most informative predictors of TADs as in Fig. 3. These significance values combined with the results above suggest the importance of the identified modifications in TADs.

#### These marks have nearly optimal coherence score

We assess the performance of various subset of modifications by the coherence score which is the exponential of the negative mean log-likelihood of each chromosome on the test set, and it is normalized by the best model coherence score as in Table 1. As such it is a relative measure of the quality of various models. The coherence score using only the set {H3K36me3, H3K4me1, H3K4me3, H3K9me3} is almost as high as the score for all 28 histone modifications in human IMR90. Restricting the effect function shape to be nonnegative and concave slightly improves the score. Similar ordering of models according to coherence scores is also observed in human ES cells as in Table 2. Our analysis indicates that the remaining modifications carry either redundant information or are less important for TADs.

**Table 1:** Normalized coherence scores of various marker subsets in human IMR90 cells

Allowed modifications (human IMR90 to IMR90)	Coherence score
	(Normalized)
28 histone modifications + Concave + Nonnegative	1.00
28 histone modifications + Concave	0.99
28 histone modifications	0.97
H3K4me3, H3K79me2, H3K27ac, H3K9me3, H3K36me3, H4K20me1	0.94
H3K36me3, H3K4me1, H3K4me3, H3K9me3 + Concave + Nonnegative	0.94
H3K36me3, H3K4me1, H3K4me3, H3K9me3 + Concave	0.93
H3K36me3, H3K4me1, H3K4me3, H3K9me3	0.92

**Table 2:** Normalized coherence scores of various marker subsets in human ES cells

Allowed modifications (human ES to ES)	Coherence score
	(Normalized)
28 histone modifications + Concave + Nonnegative	1.00
28 histone modifications + Concave	0.96
28 histone modifications	0.97
H3K4me3, H3K79me2, H3K27ac, H3K9me3, H3K36me3, H4K20me1	0.91
H3K36me3, H3K4me1, H3K4me3, H3K9me3 + Concave + Nonnegative	0.90
H3K36me3, H3K4me1, H3K4me3, H3K9me3 + Concave	0.88
H3K36me3, H3K4me1, H3K4me3, H3K9me3	0.91

### Predicting TADs from histone marks in human

**Fig. 4:**
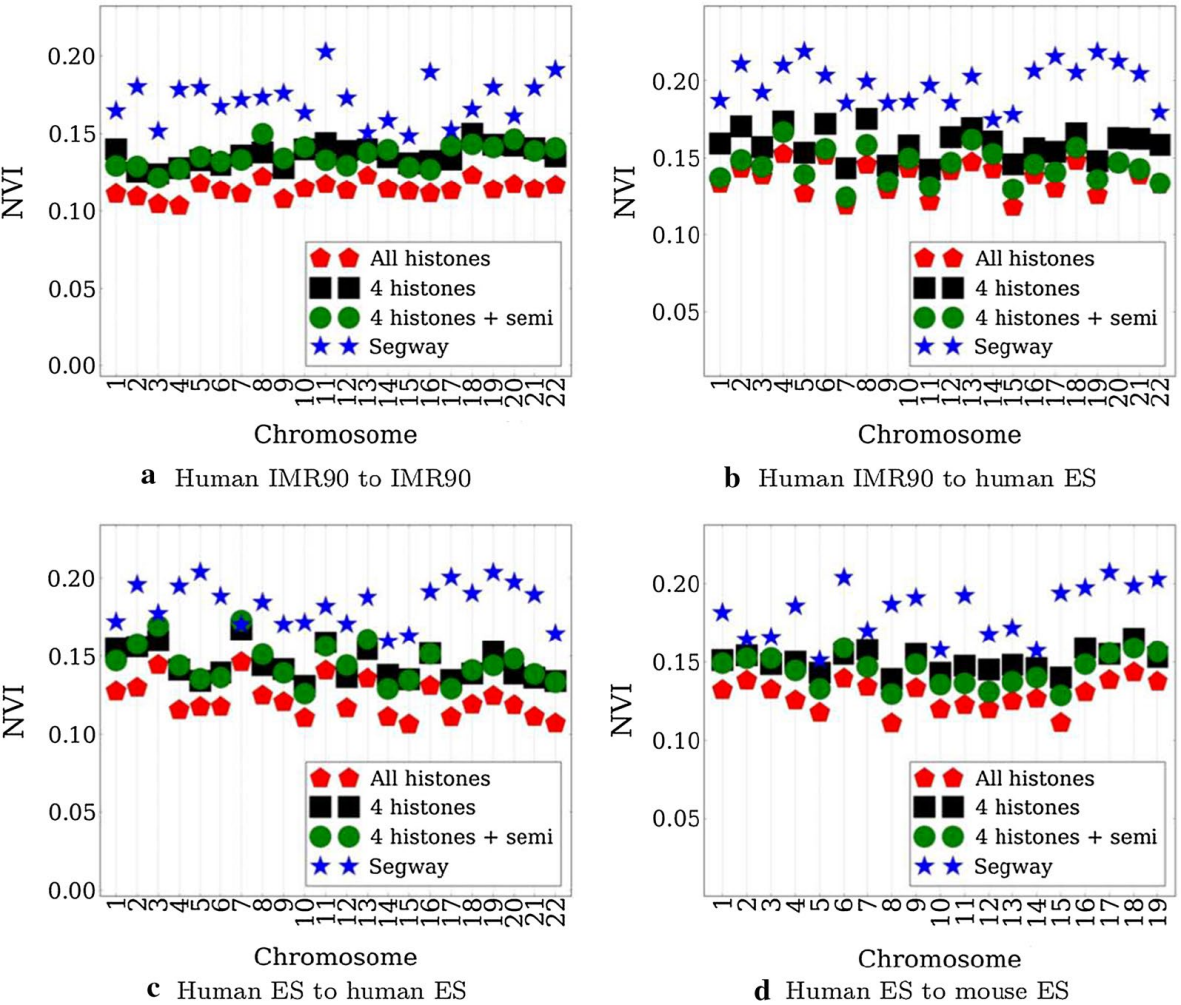
TAD prediction performance on different chromomes.** a** human IMR90 to human IMR90: infer each human IMR90 chromosome by training with all IMR90 chromosomes except the one to be inferred,** b** human IMR90 to human ES,** c** human ES to human ES,** d** human ES to mouse ES are defined similarly

*nTDP* is able to predict domain boundaries accurately using 4 histone marks alone in both human IMR90 and human ES cells. We compare TAD prediction performance of *nTDP* with the chromatin state partition predicted by Segway [16] in terms of NVI. Even though Segway does not predict TADs directly, its chromatin state partition can still be used as a baseline. Training with all 28 histone modifications instead of with the identified 4 modifications does not lead to a major performance increase as shown in Fig. 4a even though it increases the training time approximately 4 times for human IMR90 cells. Restricting the effect function to be monotonic and concave only slightly increases the performance. Chromatin states inferred by Segway do not directly correspond to TADs which leads to a lower TAD prediction performance even though they have other meaningful interpretations.

We find combinatorial effects of histone modifications to be important for accurate domain prediction since none of the modifications can achieve NVI score better than 0.2 when considered independently. To verify that there are not inherent structures in the data that can lead to an easy prediction, we randomly shuffle domains in the training set by preserving their lengths without shuffling modifications, which NVI score is never better than 0.3 in all chromosomes showing the importance of histone modification distributions in TADs.

*nTDP* also predicts TADs accurately across different species as well as across different cell types as in Fig. 4b–d. For example, if we train on human IMR90 data, the model we obtain is still able to recover domains in human ES cells (Fig. 4b). Using the identified 4 histone modifications achieves NVI score of 0.13 in human ES whereas using all modifications achieves slightly lower 0.11 on average. This performance difference is not significant except chromosomes 7 and 21 in human ES. This holds true across species as well: training on human ES data, for example, produces a model that can work well on mouse ES data. Accurate prediction of TADs by training with the identified 4 histone modifications across different species and cell types suggests the consistency of the identified modifications across species and cell types.

**Fig. 5:**
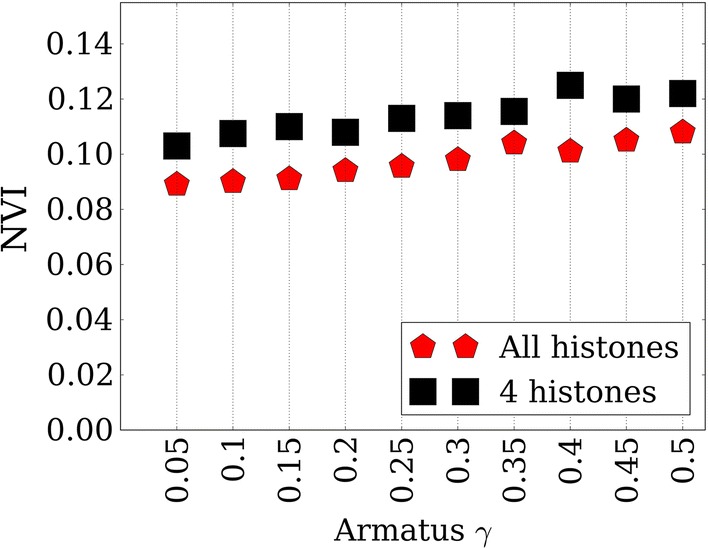
Multiscale analysis of the predicted TADs. Performance over true TAD partitions at different scales obtained via different *Armatus* $\gamma$ in human IMR90

### Multiscale analysis of the predicted TADs

We find that our predicted TADs match true TADs more accurately at different scales defined by different *Armatus* $\gamma$’s as in Fig. 5. We observe a slight performance improvement if we define true TAD partition at lower *Armatus* $\gamma$ values in human IMR90 which correspond to longer TADs. This figure suggests that some of our wrong TAD predictions may actually correspond to longer TAD blocks which we erroneously interpret as incorrect due to a scale mismatch.

## Conclusion

We formulate semi-nonparametric modeling of TADs in terms of histone modifications, and propose an efficient provably optimal solution *nTDP* for training and inference. Experimental results on human and mouse cells show that a common subset of histone modifications can accurately predict TADs across cell types and species. Via our trained model, we also accurately predict TADs without using any Hi-C data which is especially useful for understanding the 3D genome conformation on species with limited Hi-C data. We expect our method to become increasingly useful with faster accumulation of epigenomic datasets than Hi-C interaction data. Additionally, some of our mispredictions may actually correspond to TADs at different scales suggesting a possibility of better inference performance than presented here.
